# Chromium and cobalt ion concentrations in blood and serum following various types of metal-on-metal hip arthroplasties: A literature overview

**DOI:** 10.3109/17453674.2013.792034

**Published:** 2013-05-31

**Authors:** Christopher Jantzen, Henrik L Jørgensen, Benn R Duus, Sune L Sporring, Jes B Lauritzen

**Affiliations:** ^1^Departments of Orthopaedic Surgery; ^2^Clinical Biochemistry, Bispebjerg Hospital, University of Copenhagen, Copenhagen, Denmark.

## Abstract

**Background and purpose:**

Widely different metal ion concentrations in blood and serum have been reported with metal-on-metal (MoM) implants. We reviewed the literature on blood and serum ion concentrations of chromium (Cr) and cobalt (Co) following various MoM hip arthroplasties.

**Methods:**

Studies were searched for in the Medline database, Embase, and the Cochrane Database of Systematic Reviews. Highest mean or median ion concentrations of Cr and Co after a minimum of 1 year of follow-up were extracted and grouped according to sample- and articulation type, and average values were calculated.

**Results:**

43 studies were included and 16 different MoM implants were identified. For the different types of bearings, average ion concentrations and range were calculated from the mean or median ion concentration. The average Cr concentration ranged between 0.5 and 2.5 μg/L in blood and between 0.8 and 5.1 μg/L in serum. For Co, the range was 0.7–3.4 μg/L in blood and 0.3–7.5 μg/L in serum.

**Interpretation:**

When the average blood ion concentrations calculated for the different implants, together with the concentrations measured in the individual studies, were compared with the upper acceptable limit for Cr and Co in blood, no clear pattern was recognized. Furthermore, we were unable to detect any clear difference in ion concentrations between different types of implants (THA and resurfacing).

It is well known that metal-on-metal (MoM) hip arthroplasties lead to increased whole blood levels of chromium (Cr) and cobalt (Co), and there is increasing concern about the possible toxic effects: local tissue toxicity, inflammation, bone loss, impaired renal function, immune modulation, hypersensitivity, chromosomal damage, malignant cellular transformation, pseudotumor formation, and soft tissue necrosis (Merritt and Brown 1996, Jacobs et al. 2001, Signorello et al. 2001, Tharani et al. 2001, Anissian et al. 2002, Shimmin et al. 2005, Willert et al. 2005, Hart et al. 2006, Keegan et al. 2007, Mabilleau et al. 2008, Pandit et al. 2008). Causal associations between MoM bearings and the potential risks have not yet been established; nor have safe levels for metal ions (MacDonald 2004). We overviewed the current literature on blood and serum concentrations of Cr and Co following various types of MoM arthroplasties.

## Material and methods

### Search strategy

A search was conducted in the Medline database and Embase with the following keywords: (“hip prosthesis” or “total hip arthroplasty” or THA or “hip replacement” or “hip and arthroplasty” or resurfacing) and (chromium or cobalt or chrome) and (ion or ions).

**Figure 1.**
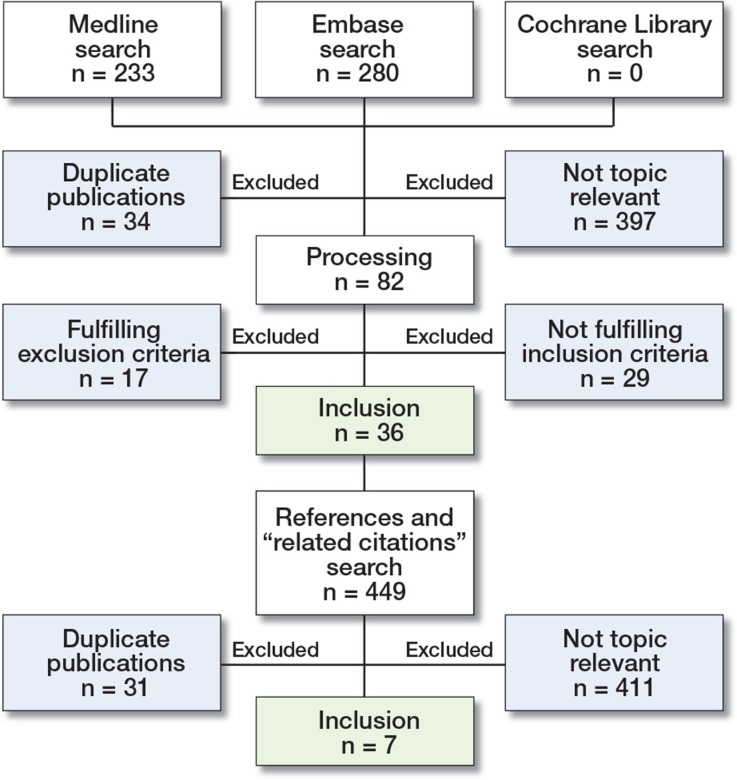
Flow diagram illustrating the study selection process.

Furthermore, we searched the Cochrane Database of Systematic Reviews for relevant articles. Titles and abstracts were screened and relevant articles were chosen for full-text review. The reference lists of these and the “related citations”-box were used to find additional studies. The search was conducted on December 13, 2012 and resulted in the inclusion of 43 studies (Figure 1). Evaluation of the studies for inclusion was conducted by 2 independent researchers. To ensure inclusion of all relevant articles and data, the search results were screened twice.

### Study selection

There were 8 inclusion criteria: 1. English text; 2. human studies; 3. studies on medically healthy patients; 4. retrospective or prospective studies with specifications of the arthroplasty type; 5. unilateral arthroplasty; 6. MoM resurfacing or total hip arthroplasty; 7. measurement of Cr and Co levels in serum or blood; and 8. a mean follow-up of at least 1 year. There were 3 exclusion criteria: 1. duplicate publications; 2. bilateral implants; and 3. single case reports.

For studies concerned with various types of MoM arthroplasties, separate measurements of Co and Cr were required for the different types. Also, if it was not possible to obtain the maximum ion concentrations of Cr and Co, the study was excluded.

### Analysis

From the studies included, we extracted the highest mean or median serum or blood ion concentrations of Cr and Co after a mean follow-up of at least 1 year. A period of at least 1 year was chosen to reduce the influence of the running-in period (Heisel et al. 2008). Values measured in moles were converted to grams by dividing the ion concentrations by atomic weight (52.00 g/mole for Cr and 58.93 g/mole for Co). The mean or median ion concentrations extracted were grouped according to type of sample and implant type. These values were then used to calculate average blood or serum ion concentrations for the separate implants. Furthermore, pertinent information was collected from each study (author, study design, type of hip prosthesis and size, number of participants, their age and sex, follow-up period, type of sample, method of analysis, highest concentrations of Cr and Co, and time of follow-up until maximum ion concentrations). Regarding study type, these were grouped as randomized clinical trials (RCTs), retrospective studies (RSs), or non-randomized clinical trials (non-RCTs)—the latter covering non-randomized prospective and longitudinal studies. Since graphite-furnace atomic absorption spectrometry (GF-AAS) and electrothermal atomic absorption spectrometry (ETAAS) are synonymous, these were grouped as atomic absorption spectrometry (AAS). In cases where the precise type of MoM implant was not clearly specified, the internet was searched for information or the manufacturer was contacted in order to clarify this. If this was not possible, the study was excluded.

## Results

The literature search resulted in the inclusion of 43 studies from which the highest mean or median Cr and Co ion concentrations after a minimum of 1 year could be obtained (Table). 16 different MoM bearings were identified including: Articular Surface Replacement (ASR; DePuy), Birmingham Hip Resurfacing (BHR; Smith and Nephew), Metasul 28-mm THA (Zimmer), Conserve Plus (Corin), Articular Surface Replacement XL (ASR XL; DePuy), Ultamet (DePuy), Cormet 2000 (Corin), Durom Hip Resurfacing (Zimmer), BHR with adapter sleeve (Smith and Nephew), Metasul Large Diameter Head (LDH) THA (Zimmer), Sikomet-SM21 (Sikov Medizintechnik GmbH), M2a-Magnum (Biomet), Optimom (Corin), Conserve Total (Wright Medical Technology), Total A-Class (Wright Medical Technology), and Ultima (Johnson and Johnson).

**Table:** Characteristics of the studies included

A	B	C	D	E	F	G	H	I	J	K
Langton 2009	– **^b^**	ASR, HRS; 49 (41–59)	90 (42)	55 (28–77)	26			26:26	3.6**^a^** (1.5–70)	2.1**^a^** (0.4–271)
		BHR, HRS; 49 (38–58)	70 (42)	51 (32–67)	47	WB	P	47:47	4.0**^a^** (2.4–40)	1.4**^a^** (0.6–147)
Yang 2011	–	Conserve plus, HRS; NA	25 (NA)	37 (24–46)	24	S	A	24:24	0.7**** (0.4–0.8)	0.4**** (0.3–0.5)
Isaac 2009	–	ASR, HRS; 53 (41–61)	77 (56)	60 (31–69)	24	WB	P	24:24	1.5**^a^** (NA)	1.5**^a^** (NA)
Heisel 2008	–	ASR, HRS; 48 (NA)	15 (7)	51 (31–61)	12	S	H	12:12	2.7**^a^** (0.5–20)	2.5**^a^** (0.4–15)
Hasegawa 2012	–	Optimom, THA, NA	75 (11)	65 (40–84)	24	S	P/A	12:12	2.1**^a^** (NA)	2.3**^a^** (NA)
Kim 2011	–	Conserve plus, HRS; NA	97 (76)	48 (18–65)	24	S	H	24:24	2.7**** (0.5–11)	1.8 (0.5–7.1)
Lavigne 2011	–	M2a-magnum, THA; NA						24:24	1.1**^a^** (0.4–14)	0.7**^a^** (0.3–13)
		Metasul LDH, THA; NA						24:24	1.3**^a ^**(0.4–6.1)	2.7**^a^** (0.4–8.3)
		ASR XL, THA; NA	144 (NA)	NA	24	WB	H	24:24	1.3**^a^** (0.2–6.2)	1.3**^a^** (0.3–7.6)
		BHR+ sleeve, THA; NA						24:24	1.9**^a^** (0.1–21)	1.9**^a^** (0.4–5.3)
Savarino 2006	–	Metasul, THA; 28 (0)	42 (23)	57 (31–79)	53	S	A	53:53	2.1 (0.1–8.6)	1.6 (0.1–7.3)
Moroni 2008	– **^b^**	BHR, HRS; 47 (NA)	20 (11)	53 (30–65)	58			24:24	2.3 (0.5–11)	1.1 (0.3–5.6)
		Metasul, THA; 28 (0)	35 (19)	60 (41–79)	56	S	A	24:24	2.0 (0.1–8.0)	1.4 (0.1–7.3)
Langton 2010	– **^b^**	ASR HRS; 49 (39–59)	418 (234)	56 (28–77)	35			35:35	4.2**^a ^**(1.5–70)	2.7**^a ^**(0.4–271)
		BHR, HRS; 48 (38–58)	155 (88)	51 (32–67)	63	WB	P	63:63	4.2**^a ^**(2.4–40)	1.8**^a ^**(0.6–147)
		ASR XL, THA; 47 (39–57)	87 (34)	67 (25–85)	41			41:41	3.7**^a ^**(2.4–22)	3.3**^a ^**(1.1–32)
Dahlstrand 2009	+	Metasul, THA; 28 (0)	28 (15)	65 (45–74)	24	S	H	12:24	1.2 (NA)	1.0 (NA)
Witzleb 2006	–	Metasul, THA; 28 (0)	60 (42)	54 (45–62)				12:24	1.6**^a^** (NA)	1.7**^a^** (NA)
	–	BHR, HRS; 50 (NA)	111 (44)	51 (46–58)	24	S	A	24:24	5.1**^a^** (NA)	4.3**^a^** (NA)
Bernstein 2011	–	Ultamet, THA; NA (40–44)	56 (48)	60 (36–74)	12	WB	P	12:12	0.5**^a^** (NA)	2.2**^a^** (NA)
Back 2005	–	BHR, HRS; NA (38–52)	16 (12)	51 (21–74)	24	S	P/A	12:12	1.5 (0.2–3.7)	0.7 (0.3–1.9)
Savarino 2002	–	Metasul, THA; 28 (0)	26 (12)	48 (30–67)	26	S	A	26:26	1.7 (0.2–6.7)	1.3 (0.3–5.3)
Clarke 2003	– **^b^**	BHR, HRS; 48 (38–54)	16 (NA)						2.6**^a^** (1.5–8.6)	2.1**^a^** (0.8–8.5)
		Cormet 2000, HRS; 48 (38–54)	6 (NA)	53 (39–68)	16	S	P	16	4.2**^a^** (1.3–6.7)	3.0**^a^** (1.2–6.9)
		Ultima, THA; 28 (0)	22 (NA)	61 (39–77)	20			20	1.0**^a^** (0.1–3.0)	1.3**^a^** (0.9–5.1)
Smolders 2011a	+	Conserve plus, HRS; 48 (42–54)	60 (36)	55 (25–65)				24:12	1.1**^a^** (0.1–8.4)	1.4**^a^** (0.6–12)
		Metasul, THA; 28 (0)	32 (20)	59 (55–75)	24	WB	P	24:24	0.6**^a^** (0.1–2.1)	1.1**^a^** (0.1–2.2)
Smolders 2011b	+	Conserve plus, HRS; 49 (NA)	38 (21)	58 (24–65)				24:12	1.2**^a^** (0.1–10)	1.3**^a^** (0.6–8.3)
		Metasul, THA; 28 (0)	33 (29)	59 (37–65)	24	WB	P	24:24	0.5**^a^** (0.1–2.1)	1.0**^a^** (0.1–4.2)
Antoniou 2008	–	Metasul, THA; 28 (0)	28 (11)	61 (32–74)					0.6**^a^** (NA)	2.6**^a^** (NA)
		Ultamet, THA; 36 (0)	58 (25)	58 (37–70)	12	WB	P	12:12	0.4**^a^** (NA)	2.3**^a^** (NA)
		ASR, HRS; NA	70 (54)	55 (33–73)					0.5**^a^** (NA)	2.4**^a^** (NA)
Vendittoli 2010	+	Metasul, THA; 28 (0)	53 (33)	51 (30–65)				24:24	1.6**** (0.8–5.7)	0.9 (0.2–4.9)
		Durom, HRS; 49 (40–58)	64 (42)	49 (25–64)	24	WB	H	24:24	1.6**** (0.4–3.7)	0.7 (0.2–2.9)
Vendittoli 2011	–	Metasul, THA; 48 (42–56)	29 (15)	50 (31–62)	12	WB	H	12:12	1.3 (0.6–3.1)	2.2 (0.3–5.6)
Lardanchet 2012	–	Metasul LDH, THA; NA	24 (10)	67 (50–79)	12	S	P/A	12:12	1.6**^a^** (1.0–2.4)	2.8**^a^** (1.3–6.6)
	–	M2a-magnum, THA; NA	23 (9)	67 (51–81)	12	S	P/A	12:12	2.5**^a^** (1.9–3.8)	2.2**^a^** (1.4–3.1)
	–	Conserve total, THA; NA	20 (11)	65 (45–77)	12	S	P/A	12:12	4.4**^a^** (1.4–6.3)	7.5**^a^** (3.6–10)
Williams 2011	–	Metasul LDH, THA; NA	31 (NA)	NA	24	S	H	24:24	2.8**^a^** (0.7–50)	4.5**^a^** (0.5–59)
	–	Durom, HRS, NA	20 (NA)	NA	24	S	H	24:24	1.1**^a^** (0.5–143)	0.8**^a^** (0.4–196)
Skipor 2002	–	Conserve plus, HRS; 48 (38–52)	25 (17)	49 (28–62)	12	S	A	12:12	1.8 (0.6–4.3)	1.1 (0.4–2.8)
Savarino 2008	– **^b^**	Metasul, THA; 28 (0)	16 (7)	54 (46–63)	121	S	A	121	0.9 (0.3–2.2)	0.7 (0.3–1.6)
Imanishi 2010	–	Ultamet, THA; 36 (0)	33 (4)	60 (41–83)	12	S	P	12:12	0.8**^a ^**(NA)	1.1**^a ^**(NA)
Daniel 2009	–	BHR, HRS; NA (50–54)	26 (26)	53 (35–74)	72	WB	H	12:12	2.4 (0.7–3.8)	1.3 (0.4–3.8)
Langton 2008	–	ASR, HRS; 49**^a ^**(41–59)	76 (49)	55 (35–74)	26	WB	P	26:26	3.4**^a^** (1.5–70)	2.0**^a^** (0.4–271)
Daniel 2006	–	BHR, HRS; NA (50–54)	26 (NA)	53 (19–67)	12	WB	H	12:12	2.4 (NA)	1.3 (NA)
	–	Metasul, THA; 28 (0)	28 (NA)	57 (39–63)	12	WB	H	12:12	1.7 (NA)	1.7 (NA)
Garbuz 2010	+	Durom, HRS; NA (50–54)	48 (43)	52 (NA)	13	S	H	24:24	0.8 (NA)	0.5 (NA)
Pattyn 2011	+	BHR, HRS; NA	20 (12)	49 (NA)	24	WB	P	12:24	1.5 (0.5–2.7)	2.1 (0.5–6.5)
	+	Durom, HRS; NA	22 (18)	53 (NA)	24	WB	P	12:24	1.0 (0.5–6.4)	1.1 (0.5–3.3)
	+	Metasul, THA; 28 (0)	10 (4)	54 (NA)	24	WB	P	12:24	1.8 (0.5–4.8)	2.0 (0.7–5.2)
Lhotka 2003	–	Sikomet-SM21, THA; 28 (0)	128 (41)	57 (NA)	42–48	WB	A	45:45	1.8 (NA)	3.4 (NA)
Allan 2007	–	Comet 2000, HRS; 47 (40–56)	35 (20)	51 (33–66)	28	S	H	12:12	5.1 (NA)	4.3 (NA)
Iavicoli 2006	–	Conserve plus, HRS; 44 (38–50)	14 (6)	66 (44–84)	15	S	P	45:14	2.9 (0.5–5.0)	14 (3.7–43)
Beaulé 2011	–	Conserve plus, HRS; 48 (NA)	26 (21)	54 (NA)	24	S	H	24:24	3.1 (0.6–11)	2.0 (0.4–7.1)
	–	A-Class BFH, THA; 48 (NA)	26 (18)	60 (NA)	24	S	H	12:12	3.0 (0.9–6.7)	5.1 (0.8–13)
Daniel 2007	–	BHR, HRS; NA (50–54)	26 (NA)	53 (29–67)	48	WB	H	12:12	2.4 (0.7–3.8)	1.3 (0.4–3.8)
Mauer-Erti 2012	–	ASR, HRS; 49 (45–51)	8 (5)	47 (33–57)	24	S	A	12:12	2.1**^a^** (0.8–9.7)	1.4**^a^** (0–3.9)
	–	ASR XL, THA; 46 (41–51)	28 (15)	52 (40–61)	24	S	A	24:24	4.6**^a^** (0.7–52)	3.6**^a^** (0.3–78)	
Milovsev 2005	– **^b^**	Sikomet-SM21, THA; 28 (0)	25 (11)	57 (36–72)	60	S	A	60:60	1.3 (0.3–6.5)	0.3 (0.1–0.9)
Desy 2011	– **^b^**	ASR, HRS; 53 (46–59)	91 (74)	53 (38–73)	37	WB	P	37:37	0.7**^a ^**(NA)	2.1**^a ^**(NA)
Daniel 2008	–	Metasul, THA; 28 (0)	20 (NA)	63 (55–75)	12	WB	H	12:12	1.7 (NA)	1.7 (NA)
	–	BHR+ sleeve, THA; NA (42–	28 (NA)	61 (37–77)	12	WB	H	12:12	1.4 (NA)	2.3 (NA)
Clarke 2003	–	Ultima, THA; 28 (NA)	22 (NA)	61 (39–77)	20	S	P	20:20	1.0**^a^** (0.1–3.2)	1.3**^a^** (0.9–5.1)
Lazennec 2009	–	Metasul, THA; 28 (0)	109 (56)	54 (30–60)	108	S	O	36:36	2.1**^a^** (1.0–3.0)	1.7**^a^** (1.0–2.5)
Holland 2012	–	BHR, HRS; 50**^a^** (42–58)	90 (74)	51 (21–68)	115	WB	P	115:115	1.7**^a^** (0.4–15)	1.7**^a^** (0.5–20)

**^a ^**Median values (else mean values) A First author and year of publication B Randomized clinical trial **^b ^**retrospective study C Hip prosthesis type; size (range) of femoral component, mm D Number of participants (male number) E Age (range) F Follow-up time, months G Sample: WB – whole bloode, S – serum H Method of analysis A – atomic absorption spectrometry H – high-resolution inductively coupled mass spectrophotometry O – inductively coupled optical emission spectrometry P – inductively coupled plasma mass spectrophotometry I Time (months) to peak ion concentration, Cr:Co J Cr level (range), µg/L K Co level (range), µg/L

**Figure 2.**
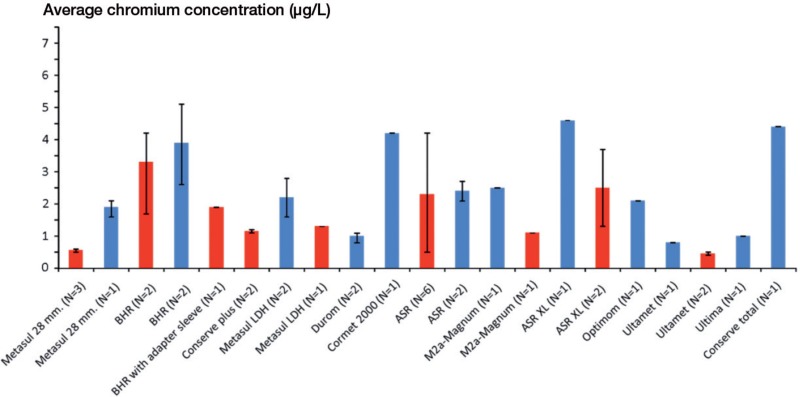
Average Cr concentration and range, calculated from medians, in blood (red) and serum (blue) following various types of MoM hip arthroplasties.

**Figure 3.**
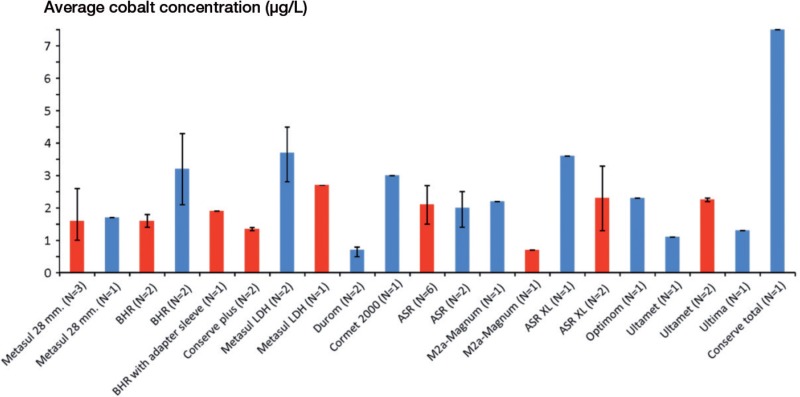
Average Co concentration and range, calculated from medians, in blood (red) and serum (blue) following various types of MoM hip arthroplasties.

**Figure 4.**
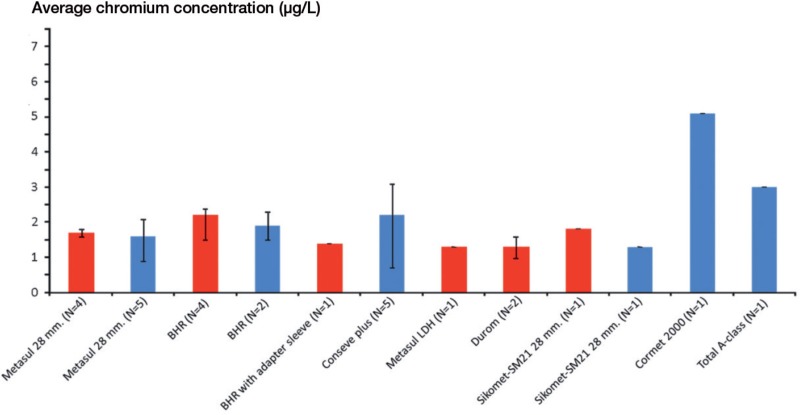
Average Cr concentration and range, calculated from means, in blood (red) and serum (blue) following various types of MoM hip arthroplasties.

**Figure 5.**
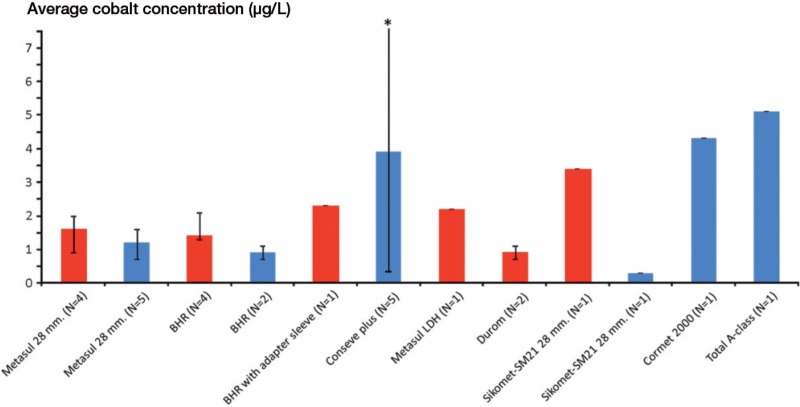
Average Co concentration and range, calculated from means, in blood (red) and serum (blue) following various types of MoM hip arthroplasties.*upper range 10.1 μg/L.

The average Cr and Co concentrations calculated from means and medians for the different implants are illustrated in Figures 2–5. Mean Co concentrations ranged between 0.9 and 3.4 μg/L in blood and between 0.3 and 5.1 μg/L in serum; median values ranged between 0.7 and 2.7 μg/L in blood and between 0.7 and 7.5 μg/L in serum. Mean Cr concentrations ranged between 1.3 and 2.2 μg/L in blood and between 1.6 and 5.1 μg/L in serum; median values ranged between 0.5 and 2.5 μg/L in blood and between 0.8 and 4.6 for μg/L in serum.

## Discussion

We could not detect any clear difference in ion concentrations between the different types of MoM THAs and MoM resurfacing systems. Precautions should be taken when interpreting our results because of the few studies dealing with different implant types. There was also a lack of consensus between the studies about whether to measure ion concentrations in serum or whole blood, and whether the values measured should be expressed as means or medians. Many of the studies included small populations, with only 4 of the 43 studies involving more than 100 patients. It should be noted that the mean/median ion concentrations extracted from the studies included were the highest ones measured after a minimum of 1 year. As such, the values used to calculate means and for the different MoM bearings were not measured at exactly the same time. The calculated values therefore represent maximum concentrations for the different implants.

Monitoring of the systemic ion exposure after implantation of MoM bearings can be done in whole blood, serum, and erythrocytes as well as in urine and other body fluids. Most studies measured the ion concentrations in either serum or whole blood. Daniel et al. (2007b) suggested that serum concentrations should not be used as surrogate measures of systemic metal ion exposure and that blood and serum values cannot be used interchangeably. This is supported by Smolders et al. (2011a) who also provided a formula for conversion between the 2 values. For studies measuring metal ion levels, an important consideration is the valency (electron charge) of these ions. This is especially important with chromium ions, since it is well established that the Cr^4+^ ion in particular is very oxidative and can easily enter cells, in contrast to the more commonly found Cr^3+^ ion. Because of this the Cr^4+ ^ion is more carcinogenic (Standeven and Wetterhahn 1991) and cytotoxic (Bagchi et al. 2002) than the Cr^3+^ ion. In the long term studies investigated, we assume that Cr^4+^ was reduced to Cr^3+^, since this happens rapidly (Rogers 1984). The valency state of Co is mostly Co^2+^ in vivo (Lauwerys and Lison 1994, Ning et al. 2002). The biological effect is therefore not only related to length of exposure but also to the ions initially generated. But even though the valency of the ions is important regarding toxic effects, none of the studies included were concerned with this and they only measured total ion concentrations.

Many concerns have been raised about the potential toxic effects of prolonged exposure to Cr and Co after insertion of MoM bearings (Merritt and Brown 1996, Jacobs et al. 2001, Signorello et al. 2001, Tharani et al. 2001, Anissian et al. 2002, Shimmin et al. 2005, Willert et al. 2005, Hart et al. 2006, Keegan et al. 2007, Mabilleau et al. 2008, Pandit et al. 2008). However, it has not yet been possible to establish a causal association between toxic effects and MoM arthroplasties. The ion concentrations at which toxicity is induced are as yet known, so safe levels of Cr and Co cannot be determined (MacDonald 2004). Based on 2 studies by Hart et al. (2008, 2009), acceptable upper limits of 2.56 μg/L for Cr and 2.02 μg/L for Co in whole blood have been proposed. 19 of the 43 studies included here, involving 11 prosthesis types, measured ion concentrations in blood. 34 measurements were made, and 6 of these exceeded this maximum value for Cr.

Of these were 3 concerned with ASR (of 6), 2 with BHR (of 6), and 1 with ASR XL (of 2). For Co, the upper limit was exceeded in 11 studies. Of these were 4 concerned with ASR (of 6), 2 with Metasul LDH (of 2), 1 with BHR (of 6), 1 with ASR XL (of 2), 1 with Metasul 28-mm THA (of 7), 1 with Sikomet-SM21 (of 1) and 1 with BHR with adapter sleeve (of 2).

When looking at the average ion concentrations calculated for the different implants, the upper limit for Cr was exceeded with the BHR, but only when looking at the average of the median values. For Co, the limit was exceeded with the Metasul LDH, the ASR, the ASR XL, and the Ultamet when looking at the average of the medians—and by the BHR with adapter sleeve, the Metasul LDH, and the Sikomet-SM21 when looking at the average of the means. It is important to remember that the upper limits are not concentrations at which toxicity is induced, since these have not yet been established.

One meta-analysis (Kuzyk et al. 2011) compared the concentrations of Cr and Co after MoM hip resurfacing and MoM THA. The mean differences in ion concentrations were not statistically significantly different between the 2 techniques, which is similar to our findings. Savarino et al. (2002) found higher Cr and Co levels in patients with MoM THAs than in patients with metal-on-polyethylene (MoP) THAs. These results are supported by a meta-analysis by Qu et al. (2011). However, the incidence of complications and the reoperation rates did not differ between prosthesis types. It has also been shown that removal of MoM implants can reduce ion concentrations to near-normal levels after 1 year (Ebreo et al. 2011).

One of the major concerns about MoM arthroplasties is the postulated increased risk of cancer. A recent study did not find any relationship between MoM bearings and increased risk of cancer during the first 7 years after surgery (Smith et al. 2012). This is supported by the work of Visuri et al. (2010) who, after 20 years of follow-up, found that the cancer-related mortality in patients with MoM arthroplasties was similar to that in the general population. The incidence of pseudotumor formation has been found to be higher in patients with MoM arthroplasties than in those with MoP arthroplasties (Williams et al. 2011). In that study, there was no significant correlation between metal ion levels and the size of the pseudotumor; nor was it possible to detect a significant difference in metal ion levels between patients with pseudotumor formation and patients without.

In the present study, we tried to reduce the confounding effect of bilateral implants and renal insufficiency by excluding studies with these patients, since these factors are known to increase ion concentrations (Hur et al. 2008, Pelt et al. 2011).

The study had some limitations. The studies included were an inhomogeneous group and for this reason the evaluation of bias was omitted. Maximum ion measurements were from different time points, and different techniques were used for metal ion measurements—not all of which were the most reliable ICP-MS. Only a few studies were concerned with the different types of bearings, and most of them were small population trials. In addition, a number of factors (e.g. acetabular inclination and anteversion) are known to influence the concentration of metal ions in patients with MoM arthroplasties (Langton et al. 2008, 2009, 2010, Desy et al. 2011, Moroni et al. 2011), all which were not covered in this study.
